# Perceptions and impact of a multisectoral nutrition-sensitive agricultural program among people living with HIV in Senegal, West Africa: A pilot study

**DOI:** 10.1371/journal.pgph.0006743

**Published:** 2026-07-08

**Authors:** Sarah Drummond, Jacques F. Sambou, Seckou Badji, Anaïs Senghor, André Sadio, Cherif Gueye, Sidy Diatta, Bokenie Diedhiou, Baboucar Diatta, Jean Phillippe Diatta, Noël Magloire Manga, Noah Barclay-Derman, Sarah Koch, Salif Sow, Boubacar Kande, Stephen E. Hawes, Geoffrey S. Gottlieb, Moussa Seydi, Noelle A. Benzekri

**Affiliations:** 1 Development in Gardening, Atlanta, Georgia, United States of America; 2 Centre de Santé de Ziguinchor, Ziguinchor, Senegal; 3 Hôpital de la Paix, Ziguinchor, Senegal; 4 Department of Biomedical and Health Sciences, University of Vermont, Burlington, Vermont, United States of America; 5 Services des Maladies Infectieuses et Tropicales, Centre Hospitalier National Universitaire de Fann, Dakar, Senegal; 6 Department of Epidemiology, University of Washington, Seattle, Washington, United States of America; 7 Department of Global Health, University of Washington, Seattle, Washington, United States of America; 8 Department of Medicine, Division of Allergy and Infectious Diseases, and Center for Emerging and Re-Emerging Infectious Diseases, University of Washington, Seattle, Washington, United States of America; 9 School of Public Health, University of Washington, Seattle, Washington, United States of America; PLOS: Public Library of Science, UNITED STATES OF AMERICA

## Abstract

Food insecurity is highly prevalent among people living with HIV (PLHIV) in Senegal and is associated with poor HIV outcomes. Multisectoral nutrition-sensitive agricultural (MNSA) programs may address interconnected drivers of poor HIV outcomes, but evidence is limited, particularly in West Africa. This pilot study evaluated the perceptions and impact of a MNSA program implemented in Senegal. We conducted a convergent mixed methods study among adults ≥18 years of age, living with HIV and receiving ART in Ziguinchor, Senegal. For the quantitative strand, data from MNSA program participants and historical controls were compared. Quantitative measures included the Household Food Insecurity Access Scale (HFIAS), Body Mass Index (BMI), CD4 cell count, and HIV viral load. Group differences were assessed using Chi-square and Mann-Whitney U tests, and logistic regression was used to examine associations with viral suppression. For the qualitative strand, semi-structured interviews were conducted with MNSA program participants. Qualitative data were analyzed thematically. Data from 56 program participants and 59 controls were included. Viral suppression (<1000 copies/mL) was significantly higher among MNSA participants than controls (94.2% versus 77.8%, p = 0.02). Participants had higher median BMI (24.8 versus 21.7, p < 0.01) and lower prevalence of underweight. Moderate-to-severe food insecurity was lower among program participants, though differences were not statistically significant. Logistic regression showed a trend towards an association between MNSA program participation and viral suppression (OR 3.84; 95% CI 0.97–15.13). Qualitative interviews revealed five major themes: greater autonomy and empowerment, improved mental health, strengthened social cohesion, improved household nutrition and financial stability, and enhanced ART adherence. Findings from this pilot study suggest that the MNSA program is acceptable and may be associated with improvements in viral suppression, nutritional status, food security, and psychosocial well-being among PLHIV. Future studies using a randomized controlled approach are needed to further evaluate impacts, explore mechanisms, and assess potential for scalability.

## Introduction

Food security is defined by the United Nations Food and Agriculture Organization as, “a situation that exists when all people, at all times, have physical, social and economic access to sufficient, safe and nutritious food that meets their dietary needs and food preferences for an active and healthy life” [1]. Our previous work has shown that the majority of PLHIV in Senegal are food insecure and that food insecurity is associated with poor HIV outcomes [2–5]. The mechanisms through which food insecurity can contribute to poor HIV outcomes are mediated through vulnerabilities along the entire HIV continuum of care. Food insecurity can contribute to increased risk of HIV acquisition through high-risk behaviors and coping strategies [6–8]. Food insecurity contributes to poor adherence to ART when hunger and lack of food prevent individuals from taking their medications [2,9–12], when they are confronted with the competing demands of paying for transportation costs and medical expenses or purchasing food [9,13,14], or when time spent earning wages or performing agricultural work competes with time required to travel to the clinic and wait for care. As a barrier to ART adherence [15,16] and retention in care, food insecurity contributes to virologic failure [17], which increases risk of HIV transmission, drug resistance, and death.

The high prevalence of food insecurity among PLHIV and its role as a driver of poor outcomes across the HIV care cascade, suggesting that strategies to address food insecurity will be critical to achieving the 95-95-95 targets in Senegal. Importantly, the development and implementation of effective strategies to address food insecurity must focus on empowering women, who are not only disproportionately impacted by the HIV epidemic in sub-Saharan Africa, but are disproportionately impacted by food insecurity in every region of the world [1,18,19].

Multisectoral nutrition-sensitive agricultural (MNSA) programs are agricultural interventions that utilize cross-sectoral approaches to address the underlying determinants of nutritional status, such as food security, resource availability at the maternal, household, and community levels, access to health services, a safe and hygienic environment, and reduced vulnerability to shocks [20,21]. This study was conducted to evaluate the perceptions and impact of a MNSA program among people living with HIV in Senegal.

## Methods

### Study design

We conducted a convergent mixed methods study among adults ≥18 years of age, living with HIV and receiving ART in Ziguinchor, Senegal.

### Study site

This study took place at the Centre de Santé de Ziguinchor, located in the Casamance Region of Senegal.

### MNSA program participants

This study was conducted among a subset of individuals participating in the MNSA program. Individuals ≥18 years of age, participating in the MNSA program, and receiving ART at the Centre de Santé de Ziguinchor were eligible for enrollment in the study. Eligible participants were informed of the study by the President of the Association of People Living with HIV who is also a member of the MNSA program team. Potential participants were subsequently referred to the clinic social workers for enrollment in the study. The recruitment period was from 17/01/2023 to 27/02/2024.

### Historical controls

Historical controls were individuals ≥18 years of age who received ART for ≥12 months at the Centre de Santé de Ziguinchor, and who participated in our prior cohort study focused on food insecurity among people living with HIV in Senegal [4,5]. Data were collected from 2018 to 2019. Authors accessed de-identified historical control data for analysis in July 2024.

### MNSA program

The MNSA program was originally developed in Senegal by the non-profit organization DIG (Development in Gardening) and is based on the United Nations Food and Agriculture Organization’s (FAO) Farmer Field School training model [22]. It has since been adapted to reflect local needs and input from farmers and experts in agriculture, nutrition, public health, and small-business skills. The core objectives of the MNSA program are to improve food security, to improve the health and nutritional status of participants, and to promote social cohesion, livelihood development, and climate resilience for marginalized people. The program was implemented in the Casamance Region of Senegal in 2019.

The MNSA program begins with applied training in nutrition-sensitive, sustainable agricultural techniques using demonstration gardens that are established, maintained, and shared by program participants. The land for each demonstration garden site is provided through agreements with community leaders and participating clinics that have available land and reliable access to water from a well or a tap. DIG works with local partners, including village chiefs and health centers, to identify health posts serving marginalized communities, with a focus on women, PLHIV, and households with malnourished children. Community members are informed of the program through community and clinic-based outreach activities, and meetings held by the village chief or the lead community nurse. PLHIV specifically are informed of the program through clinic-based outreach and by the members of the Association of PLHIV.

The MNSA program is administered over the course of 12 months. Phase 1 is the intensive phase of the training program. It consists of a series of 21 modules conducted weekly at the garden site over the course of 6 months. During Phase 2, facilitators meet with the group biweekly at the garden site to provide technical assistance and support. Facilitators additionally conduct household visits to assist with the establishment and success of household gardens. Throughout the training program, multiple demonstrations are held that focus on nutrition-sensitive culinary training and community strengthening. This includes practical cooking demonstrations that use vegetables harvested from the group garden to prepare meals inspired by local, traditional recipes. The demonstrations help to introduce certain nutrient-rich vegetables and teach techniques for preparing vegetables that preserve nutritional content.

### Study procedures

Data were collected during a single study encounter using an enrollment questionnaire, validated instruments, laboratory analyses, chart review, and a semi-structured interview.

### Enrollment questionnaire

Demographic factors, socioeconomic factors, and other social determinants of health were captured using an enrollment questionnaire.

### Food insecurity

The Household Food Insecurity Access Scale (HFIAS) was used to measure food insecurity [23]. Food secure was defined as HFIAS category 1 and food insecure was defined as HFIAS categories 2–4, where category 2 is mildly food insecure, category 3 is moderately food insecure, and category 4 is severely food insecure.

### HIV outcomes

CD4 cell count and HIV viral load were measured at study enrollment. Viral suppression was defined as a viral load <1000 copies/mL. Additional HIV-associated factors were obtained from chart review.

### Statistical analysis

Descriptive analysis was performed for all quantitative variables. Absolute number and frequency were determined for categorical variables and median and interquartile range (IQR) were determined for continuous variables. The Chi-square test and the Mann-Whitney U test were used to identify differences between groups. Logistic regression was used to evaluate the association between participation in the MNSA program and viral suppression. Missing data were excluded from analysis. P-values < 0.05 were considered significant. Data were analyzed using SPSS Statistics 29.

### Semi-structured interviews

Interviews were conducted with MNSA program participants to explore perceptions of the program. Interviews were conducted by a clinic social worker using a semi-structured interview guide. Responses were recorded by the social worker using detailed notes. Detailed interview notes were uploaded into Atlas.ti (v23.4) and analyzed using thematic analysis and inductive coding [24].

### Integration

Integration of the quantitative and qualitative strands occurred at the design level, the methods level, and the interpretation and reporting level [25]. At the design level, we used the convergent mixed methods study design whereby the quantitative and qualitative strands occur in parallel and the intent of integration is to expand understanding and provide complementary results [26]. At the methods level, merging occurred by bringing the quantitative and qualitative data together for analysis and comparison. At the interpretation and reporting level, integration occurred through narrative using the weaving approach [25,27].

### Ethical statement

Participation in the MNSA program was voluntary. All study participants were informed about potential benefits and harms related to study participation. Written informed consent was obtained from all participants prior to enrollment. Consent procedures were conducted by a social worker. Study procedures were approved by the University of Washington Institutional Review Board and the Senegal Comité National d’Ethique pour la Recherche en Santé. Additional information regarding the ethical, cultural, and scientific considerations specific to inclusivity in global research is included in the Supporting Information (S1 Checklist).

## Results

Data from 56 MNSA program participants and 59 historical controls were included in this study (Table 1). There was no overlap between groups. MNSA participants were older than the historical controls, with a median age of 47.5 years (IQR 39.0–56.0, *p* < 0.01) versus 36.5 (IQR 30.0–46.8). The MNSA participants were nearly all female (98.2%) compared to the historical controls (71.2%). The proportion of participants without any formal education was similar (32.1% versus 39.7%, *p* = 0.40), as was median household size (7.5 [IQR 6.0–10.0] versus 7 [IQR 5.0–15.5], *p* = 0.64). MNSA program participants had a median of three children (IQR 2.0–6.0) while the historical controls had a median of two children (IQR 1.0–4.5, *p* < 0.01).

**Table 1:** Characteristics of people living with HIV and receiving ART in Ziguinchor, Senegal.

	MNSA program participants (n = 56)	Historical controls (n = 59)	p-value
**Age, median years (IQR)**	**47.5 (39.0**–**56.0)**	**36.5 (30.0**–**46.8)**	**<0.01**
**Female**	**55 (98.2)**	**42 (71.2)**	**<0.01**
**No formal education**	18 (32.1)	23 (39.7)	0.40
**Number of children, median (IQR)**	**3 (2.0**–**6.0)**	**2 (1.0**–**4.5)**	**<0.01**
**Number of household members, median (IQR)**	7.5 (6.0–10.0)	7 (5.0–15.5)	0.64
**Daily household food expenditure (USD), median (IQR)**	3.51 (1.76–4.39)	1.76 (1.76–3.51)	0.07
**Practice agriculture** (for MNSA program participants, prior to enrollment in the program)	**33 (60.0)**	**14 (26.9)**	**<0.01**
**Own livestock**	18 (32.1)	25 (42.2)	0.34
**Practice fishing**	0 (0)	4 (6.9)	0.12
**Own cellphone**	**53 (98.1)**	**44 (74.6)**	**<0.01**
**Household electricity**	**48 (88.9)**	**39 (66.1)**	**<0.01**
**Household tap water**	**33 (61.1)**	**20 (33.9)**	**<0.01**
**Transport time to clinic (minutes), median (IQR)**	20 (10–30)	20 (14–30)	0.38
**CD4 cell count, median cells/mm**^**3**^ **(IQR)** ^**a**^	266 (124.5–583)	378 (178–647.5)	0.34
**CD4 cell count <200 cells/mm** ^**3 a**^	12 (41.4)	10 (27.0)	0.22
**Viral suppression** ^**b**^	**49 (94.2)**	**35 (77.8)**	**0.02**
**Shared HIV status**	30 (57.7)	24 (45.3)	0.20
**BMI, median (IQR)**	**24.8 (21.2**–**28.8)**	**21.7 (19.4**–**25.5)**	**<0.01**
**BMI category**			**0.01**
<18.5	**3 (6.7)**	**8 (16.3)**	
18.5–24.9	**20 (44.4)**	**29 (59.2)**	
25.0–29.9	**14 (31.1)**	**9 (18.4)**	
≥30.0	**8 (17.8)**	**3 (6.1)**	
**Food insecurity**			0.58
Not food insecure	27 (50.0)	22 (41.5)	
Mildly food insecure	2 (3.7)	2 (3.8)	
Moderately food insecure	11 (20.4)	17 (32.1)	
Severely food insecure	14 (25.9)	12 (22.6)	
**Moderate-to-severe food insecurity**	25 (46.3)	29 (54.7)	0.38

Notes: ^a^ CD4 cell counts were available for 66 participants (29 MNSA program participants and 37 historical controls).

^b^ Viral loads were available for 97 participants (52 MNSA program participants and 45 historical controls).

Median daily household food expenditure was higher among the MNSA program participants (3.51 USD, IQR 1.76–4.39) than among historical controls (1.76 USD, IQR 1.76–3.51, *p* = 0.07). A higher proportion of MNSA participants practiced agriculture (60% versus 26.9%, *p* < 0.01). Livestock ownership was similar (32.1% versus 42.2%, *p* = 0.34). None of the MNSA participants practiced fishing, while four of the historical controls (6.9%) did. A higher proportion of MNSA program participants reported owning a cellphone (98.1% versus 74.6%, *p* < 0.01), having access to electricity (88.9% versus 66.1%, *p* < 0.01), and having access to tap water (61.1% versus 33.9%, *p* < 0.01). Median transport time to the clinic was 20 minutes for both groups (IQR 10–30, IQR 14–30, *p* = 0.38).

The median CD4 cell count was 266/mm^3^ (IQR 124.5–583) among the MNSA participants and 378/mm^3^ (IQR 178–647.5) among the historical controls (*p* = 0.34). 41.4% of the MNSA participants had a CD4 cell count <200 cells/mm^3^, compared to 27% of the historical controls, although this difference was not statistically significant (*p* = 0.22). Viral suppression was significantly higher (94.2%, n = 49) among MNSA program participants compared to historical controls (77.8%, n = 35, *p* = 0.02). A similar proportion of MNSA program participants had shared their HIV status (57.7%) compared to historical controls (45.3%, *p* = 0.20).

The median Body Mass Index (BMI) was higher among MNSA program participants (24.8, IQR 21.2–28.8) compared to historical controls (21.7, IQR 19.4–25.5, *p* < 0.01). This difference remained significant when the analysis was restricted to female participants (p = 0.005). BMI distribution differed between groups: 6.7% of MNSA participants versus 16.3% of historical controls were underweight (<18.5), 44.4% versus 59.2% were normal weight (18.5–24.9), 31.1% versus 18.4% were overweight (25.0–29.9), and 17.8% versus 6.1% were obese (≥30, *p* = 0.01).

Moderate-to-severe food insecurity among MNSA participants was 46.3% compared to 54.7% among historical controls (p = 0.38). According to HFIAS categories, 50.0% of MNSA participants and 41.5% of controls were food secure; 3.7% versus 3.8% were mildly food insecure; 20.4% versus 32.1% were moderately food insecure; and 25.9% versus 22.6% were severely food insecure (p = 0.58).

Univariate logistic regression results assessing factors associated with viral suppression are presented in Table 2. Participation in the MNSA program was marginally associated with greater odds of viral suppression (OR 3.84; 95% CI 0.97–15.13; *p* = 0.055). Age, education, food insecurity, and food expenditure were not associated with viral suppression.

**Table 2:** Univariate logistic regressions: associations of participant characteristics with viral suppression.

	OR	95% CI		p-value
**MNSA program participation**	**3.838**	**0.974**	**15.134**	**0.055**
**Age (years)**	0.981	0.934	1.031	0.452
**Lack of education**	0.761	0.219	2.647	0.667
**Moderate-to-severe food insecurity**	0.554	0.148	2.077	0.381
**Food expenditure (USD)**	1.000	1.000	1.001	0.383

### Semi-structured interviews

Five themes emerged from the interviews (Table 3).

**Table 3:** Themes and quotes from semi-structured interviews with MNSA program participants.

Theme 1. Autonomy and empowerment	“We are capable of taking care of ourselves”, “I am self-sufficient”, “I feel proud”, “I am respected by others”, “It helps people living with HIV to find autonomy”; “It gives you new knowledge”, “You learn new techniques and can teach others”, “It helps you to have more relations with others who now come to buy vegetables”
**Theme 2. Positive impacts on mental health**	“It makes you feel like a human being”, “You can see the future with hope”, “It gives you a future where you are able to improve the conditions of your own life”
**Theme 3. Social cohesion and community building**	“I have a stronger connection with my community”, “We have more opportunities because we work together”, “It improves relationships between couples”, “It builds community”, “It gives you new friendships”
**Theme 4. Benefits for the family and household**	
**Theme 4A. Enhanced food security and nutrition**	“You are able to nourish yourself better”, “It helps me to always have food for my family”, “It helps fight against childhood hunger”, “It improves nutrition for my whole family”;
**Theme 4B. Financial benefits**	“You are able to earn money and have savings”, “I can buy clothes for my children”, “I am able to take care of my family”
**Theme 5. Supporting adherence to ART**	“It helps you to come to your appointments”, “It helps you to take your antiretroviral medicine”, “It reinforces us and provides advice about treatment”

*Theme 1. Autonomy and empowerment:* A dominant theme throughout the interviews was that as a result of the program, participants experienced an enhanced sense of autonomy and empowerment. Participants said that because of participation in the program, “we are capable of taking care of ourselves”, “I am self-sufficient”, “I feel proud”, “I am respected by others”, “it helps people living with HIV to find autonomy”. The program provided women with opportunities to build new relationships through their more empowered roles: “It gives you new knowledge”, “You learn new techniques and can teach others”, “It helps you to have more relations with others who now come to buy vegetables”.

*Theme 2. Positive impacts on mental health:* Participants described how participation in the program improved their sense of self-worth and gave them hope: “it makes you feel like a human being”, “you can see the future with hope”, “it gives you a future where you are able to improve the conditions of your own life”.

*Theme 3. Social cohesion and community building:* They described the impact on social cohesion and community building: “I have a stronger connection with my community”, “we have more opportunities because we work together”, “it improves relationships between couples”, “it builds community”, “it gives you new friendships”.

*Theme 4. Benefits for the family and household:* Participants described impacts of the program on personal nutrition and food security, emphasizing the benefits for the family and household: “You are able to nourish yourself better”, “It helps me to always have food for my family”, “it helps fight against childhood hunger”, “it improves nutrition for my whole family”. Similarly, they described the financial benefits they experienced as a result of the program and emphasized the impact on their families: “You are able to earn money and have savings”, “I can buy clothes for my children”, “I am able to take care of my family”.

*Theme 5. Supporting adherence to ART:* Importantly, participants described how the program helps them with adherence to ART: “It helps you to come to your appointments”, “It helps you to take your antiretroviral medicine”, “It reinforces us and provides advice about treatment”.

## Discussion

The results of this pilot study suggest that participation in the MNSA program may have the potential to improve HIV outcomes, food security, and nutritional status, while also providing psychosocial benefits across the individual, household, and community levels. The MNSA program was also highly acceptable to participants. To our knowledge, this study is the first to evaluate the perceptions and impact of a MNSA program among PLHIV in West Africa.

Notably, the prevalence of **viral suppression** was higher among participants in the MNSA program compared to historical controls. This is consistent with our qualitative findings that the program supports adherence to ART. Our study is among the first to suggest an association between participation in MNSA programs and viral suppression [28–30]. Our hypothesis is that participation in the MNSA program helps to address potential barriers to ART adherence, including food insecurity, financial constraints, and poor mental health, thereby improving virologic outcomes.

Improved **food security** emerged as a major theme among program participants in the qualitative interviews. Participants described two primary pathways through which the program improved food access: consumption of vegetables from the garden and increased disposable income from vegetable sales. Although these improvements were not detected using the HFIAS, future studies involving a larger sample size may more reliably capture these impacts.

Participation in the program was also associated with a higher BMI and a lower prevalence of undernutrition. Of note, this difference persisted when analysis was restricted to female participants. Our study is among the first to explore whether participation in MNSA programs may be linked to improved nutritional status among PLHIV, an outcome more commonly examined in studies of food support or nutrient supplementation [31]. Qualitative accounts further reinforced this pattern, as participants consistently described enhanced nutrition as a major benefit of the program. Importantly, many participants emphasized that improvements in nutrition and food security extended to their entire household, suggesting that MNSA programs may generate benefits beyond individual outcomes. Assessment of household-level nutritional status would strengthen our understanding of the broader impacts of MNSA programs.

Differences in nutritional status may also be associated with the trend towards higher socioeconomic status (SES) among program participants. However, of note, a 2019 study in Senegal found that despite an association between higher SES and obesity/overweight, the stronger risk factors were gender (female), age (older), and the social valorization of overweight/obesity [32]. Future MNSA programs should ensure that nutrition trainings include health education about underweight, as well as overweight and obesity.

In the semi-structured interviews, participants also emphasized **increased income, savings, and spending power** as a result of the program, which likely contributed to the potential gains in food security. This aligns with the quantitative data, which shows a trend toward higher daily household food expenditure among program participants and increased cellphone ownership. While program participants also had greater access to electricity and tap water, this may be a result of the expansion of public services in the time between data collection for the two groups rather than a change in socioeconomic status [33].

**Empowerment and autonomy** emerged as dominant themes in the semi-structured interviews. Participants frequently described themselves as self-sufficient and respected by others. The source of this respect was the new knowledge and skills gained through the program, which caused others to come to buy vegetables or learn techniques from the participants. This aligns with program monitoring data suggesting that MNSA participants train an average of four others [34]. Future studies should complement qualitative methods with standardized, women-centered indices of empowerment, such as the Women’s Empowerment in Agriculture Index (WEAI).

Positive impacts on **mental health** were another recurring theme. Participants reported feeling more hopeful, self-confident, and “humanized” as a result of participation. These improvements appear to stem from greater autonomy, social connection, and food security. Future research should build on these findings by incorporating validated measures of mental health, such as the Patient Health Questionnaire (PHQ-9) [35,36].

**Social cohesion** also emerged as a major theme, with participants describing a strengthened sense of belonging to their group and broader community. This may be attributed to the MNSA program’s emphasis on shared learning, collective ownership, and self-governance within garden groups. Participants described building community ties through the sale and exchange of vegetables and through sharing agricultural knowledge with others. These experiences of enhanced standing among the community appear to be key mechanisms of the empowerment and mental health outcomes noted above.

### Prior studies

Our findings are consistent with a wider literature showing that livelihood interventions can reduce food insecurity and increase income for PLHIV and, in some cases, contribute to improved HIV and psychosocial outcomes [37–40]. For example, the MNSA program shares a number of impact pathways with Shamba Maisha, a multisectoral agricultural intervention in western Kenya [28]. An important difference is that the Shamba Maisha program required participants to have access to arable land and reliable water for irrigation, which may have excluded some of the most economically vulnerable individuals [28,29]. In contrast, the MNSA program in Senegal leveraged public and communal assets to enable the participation of individuals without private land or water access. While the Shamba Maisha pilot suggested high odds of viral suppression and a tendency toward improved BMI among the intervention cohort, this was not replicated in the cluster randomized controlled trial (RCT) [28,29]. Our qualitative findings align with Shamba Maisha, which found improvements in mental health, social cohesion, and community connectedness as well as a reduction in internalized, anticipated, and enacted stigma [41,42]. While our study did not assess stigma in a quantitative manner, our qualitative findings appear to support the theory that MNSA interventions can reduce stigma through positive changes in self-evaluation and increased social capital [43].

The multisectoral pilot intervention ProMeSA (Proyecto para Mejorar la Seguridad Alimentaria/Project to Improve Food Security) in the Dominican Republic provides another relevant comparison [44]. The intervention shared multiple components with our MNSA program in Senegal: urban gardening, comprehensive nutrition education (including garden-based cooking demonstrations), low barriers to participation, and use of community gardens. ProMeSA differed in its stronger focus on peer nutritional counseling, while the MNSA program in Senegal placed greater emphasis on entrepreneurship, value addition, and marketing. ProMeSA’s pilot cluster RCT showed preliminary evidence of improvements in food security, ART adherence, and viral suppression, as well as reductions in internalized and experienced HIV stigma [45,46]. As with the MNSA program in Senegal, participants rated the urban gardens as helpful in keeping their clinic appointments and taking medications as prescribed [44].

Additional studies, including a longitudinal study in western Kenya and a cluster RCT in southern Uganda, found that participation in microfinance initiatives was associated with increased viral suppression [30,47]. Qualitative data from several studies in Uganda, Kenya, Malawi, and Zambia also show that participation in poultry and crop farming projects is associated with enhanced social integration, improved self-esteem, and reduced stigma [37,38,48].

Taken together, our findings contribute to a growing body of literature suggesting that MNSA programs – particularly those that combine agricultural training with comprehensive nutrition and financial components – may offer a powerful pathway towards improving HIV outcomes as well as food security, nutritional status, mental health, and social cohesion. Specifically, our study is among the first to evaluate the association between participation in MNSA programs and viral suppression, and the first to do so in a West African context.

### Limitations

The most important limitation of our study was that participants were not randomized, and the comparison group consisted of historical controls. The two cohorts were not contemporaneous as enrollment and data collection were interrupted by the COVID-19 pandemic, so differences in outcomes may partially reflect broader improvements in HIV care, such as the rollout of tenofovir-lamivudine-dolutegravir (TLD)-based regimens, or improvements in social services over time. Due to this limitation, we cannot infer a causal relationship between participation in the program and outcomes. The small sample size also limited statistical power and precluded multivariate analysis, particularly as viral load data were not available for all participants. In addition, perspectives of the control group were not captured, thus limiting our ability to compare perceptions between groups.

While women make up a majority of PLHIV in Senegal, they were overrepresented among the MNSA program participants, with only one male opting to join the program [49]. The implementing team hypothesizes that this may be due to the cultural perception of vegetable gardening as a women’s activity, particularly when occurring at a home rather than commercial level. This is consistent with research across sub-Saharan Africa describing home gardens as a women’s domain, especially when production is intended for household consumption [50–52]. While this means that findings may not hold true for men, it also gives us important insight into the impact of MNSA programs on female PLHIV, as few studies provide sex-disaggregated effects on key quantitative outcomes such as HFIAS and virologic suppression [53]. Beyond gender, other baseline differences between the two groups may introduce confounding, including age, number of children, cellphone ownership, and access to electricity and tap water. Despite these important limitations, there were compelling differences in outcomes between the two groups that warrant evaluation using a randomized controlled study approach.

## Conclusion

The findings from our pilot study support the acceptability of the intervention and suggest that participation in the program may be associated with improvements in mental health, food security, nutrition, and viral suppression. The program also appeared to strengthen participants’ sense of empowerment and connection to their wider community, highlighting the potential of integrated, multisectoral approaches to simultaneously increase viral suppression and enhance the overall well-being of PLHIV. Future studies using a randomized controlled approach are needed to confirm impacts, further explore mechanisms, and assess scalability within West African health and agricultural extension systems.

## Supporting information

S1 Checklist Inclusivity in global research. (DOCX)
